# The effect of the swaddling method on stress levels in newborns administered nasal CPAP

**DOI:** 10.1186/s12887-023-04457-5

**Published:** 2023-12-12

**Authors:** Zehra Akkoca, Betul Yavuz, Ayşe Koçak Sezgin, Yaşar Bildirici

**Affiliations:** 1 Neonatal Intensive Care Unit, Eskisehir City Hospital, Neonatal Nurse, Eskisehir, Turkey; 2 Faculty of Health Sciences, Department of Pediatric Nursing, Kütahya Health Sciences University, Kutahya, Turkey; 3 Faculty of Medicine, Basic Medical Science, Medical Biochemistry Department Kutahya, Kütahya Health Sciences University, Kutahya, Turkey; 4 Department of Pediatrics Eskisehir, University of Health Sciences, Eskisehir City Hospital, Eskisehir, Turkey

**Keywords:** Nasal CPAP, Newborn, Swaddling, Stress

## Abstract

**Background:**

This study aims to investigate the effects of the swaddling method on the stress levels in newborns receiving nasal continuous positive airway pressure (nCPAP).

**Methods:**

The study was conducted between 1 June 2022 and 1 October 2022 with 40 newborns who underwent nCPAP in the second-level Neonatal Intensive Care Unit (NICU) of a city hospital in the Central Anatolia Region of Turkey. Data were collected using a descriptive form, including the characteristics of newborns, a patient follow-up chart, and the Newborn Stress Scale (NSS). The descriptive form, the patient follow-up chart, and the NSS were completed by the researcher 30 min after the nCPAP was started and the first saliva sample was taken. The patient follow-up chart and NSS were completed 30 min after applying the swaddling method and the second saliva sample was collected. The data were analyzed using IBM SPSS Statistics 25.0 package software and presented with number, percentage, mean, standard deviation, min-max, and t-test.

**Results:**

The study found that the mean score of the NSS after the intervention (3.52 ± 2.57) was lower than that before the intervention (10.02 ± 2.05) (*p* < 0.05). The mean saliva cortisol levels of the newborns after the intervention (4.99 ± 1.89) were lower than before the intervention (5.51 ± 1.65) (*p* < 0.05). The mean heart (135.50 ± 14.15) and respiratory rates (68.07 ± 10.16) of the newborns after the intervention were lower than those before the intervention (140.82 ± 18.11; 72.95 ± 9.06, respectively) (*p* < 0.05). There was no difference between the mean oxygen saturation of newborns before and after the intervention (*p* > 0.05).

**Conclusions:**

The study showed that the swaddling method played a role in reducing the stress levels in newborns who underwent nCPAP. It is recommended that randomized controlled trials examining the effect of swaddling on the stress levels of newborns who underwent nCPAP be conducted.

## Background

Nasal continuous positive air pressure (nCPAP), one of the noninvasive mechanical ventilation methods, is used in patients with spontaneous breathing and when free oxygen therapy is not sufficient [1]. Thus, nCPAP improves impaired gas exchange by providing a continuous flow of positive pressure to prevent alveolar collapse [2]. nCPAP is the treatment of choice for bronchopulmonary dysplasia (BPD), respiratory distress syndrome (RDS), meconium aspiration syndrome (MAS), pulmonary hypertension, pneumonia, as well as transient tachypnea of newborn (TTN) [1].

Nasal cannulas used during nCPAP administration to newborns may irritate their nasal mucosa and nasal septum [3]; nasal masks may compress the nasal root and nasal circumference [4], and excessive pressure may cause gas accumulation in the abdomen and stomach [5]. In addition to the respiratory stress, nCPAP application may cause newborns to experience stress due to its undesirable effect (nasal travma) [6]. In the literature, it is recommended to use pharmacological and non-pharmacological methods (oral sucrose/glucose, breast milk, pacifier, kangaroo care, flexion posture, swaddling, reducing environmental stimuli) to reduce the pain and stress levels experienced by newborns in interventions applied to newborns in neonatal intensive care units (NICU) [7–9]. When premature babies are wrappeded, they show improved neuromuscular development, less physiological distress, better motor organization, and greater self-regulation ability [10]. Swaddling is a nonpharmacological method that can be used frequently in NICUs because it reduces the stress caused by simple, safe, moderate, and low interventional pain [8, 11]. Swaddled term and preterm infants wake up less and experience less physiological distress and stress [12]. In the literature, studies using the Premature Infant Pain Profile (PIPP) [11, 13–15] and Neonatal Infant Pain Scale (NIPS) [16–19] to evaluate pain have reported that swaddling reduces pain and therefore stress. However, no investigation has examined the effect of the swaddling method on the stress levels of newborns undergoing nCPAP. Based on the literature review, it was thought that the swaddling method during nCPAP application may have a positive effect on reducing the stress levels of newborns and this application can be easily applied by nurses providing care in NICUs.

To date, no investigation has examined the effect of the swaddling method on the stress level of newborns undergoing nCPAP.

Study hypotheses:

### H_1_

Swaddling of newborns receiving nCPAP has an effect on the stress score.

### H_2_

Swaddling of newborns receiving nCPAP has an effect on saliva cortisol levels.

## Methods

### Design and sample

**Fig. 1:**
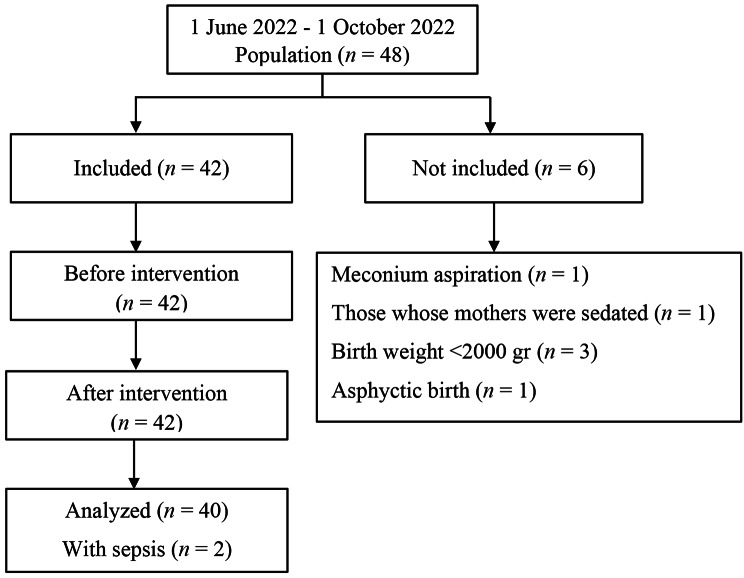
Flowchart of the study

This study was designed in a single group, pretest-posttest experimental type. The study was conducted in the second-level NICU of a city hospital in the Central Anatolia Region of Turkey between 1 June 2022 and 1 October 2022. The study included 42 newborns who underwent nCPAP in the NICU. Of the patients, two were excluded from the sample group because they developed sepsis, and the study was completed with 40 newborns (Fig. 1).

The inclusion criteria were as follows: (1) Newborns with a gestational age of 35 weeks or more; (2) a birth weight of 2,000 g or more (there is no consensus on when the cortisol circadian rhythm is established or whether it depends on gestational age or postnatal age [20]); (3) had a diagnosis of TTN; (4) been decided to administer nCPAP; (5) whose parents agreed to participate in the study.

The exclusion criteria were as follows: (1) Newborns whose mothers took cortisol-containing drugs in the antenatal period, used addictive substances in the antenatal period; (2) newborns with chorioamniotic and metabolic disease (adrenal insufficiency, etc.) (3) newborns whose amniotic fluid was stained with meconium, who were intubated, (4) whose APGAR score was < 6, (5) who were administered analgesic or anesthetic drugs for sedation, who were administered cortisol-containing drugs, (6) whose saliva sample could not be obtained or was contaminated with blood, (7) who had signs of nasal injury during nCPAP, (8) who had congenital defects that prevent the swaddling method, (9) who were administered resuscitation, (10) who were asphyctic at birth, who had non-respiratory causes [21, 22], (11) neonatal sepsis, or hypocalcemia were not included in the study.

#### Variables of the study

The independent variable of the study was the swaddling method; the dependent variables were the stress score of the newborn and the saliva cortisol levels.

### Data collection

#### Newborn descriptive form

This form was prepared by the researchers in line with the literature on the subject. The form includes information about the newborn’s age, height, head circumference, birth weight, birth week, mode of delivery, APGAR score, gender, nasal cannula and nasal mask use in nCPAP application, invasive intervention applied, and mother’s age [23].

#### Inclusion and exclusion Criteria Form

This form includes inclusion and exclusion criteria in order to determine the sample.

#### Patient follow-up table

This table includes vital signs (heart rate, oxygen saturation, respiratory rate) and cortisol level at the time the saliva sample was taken.

*Newborn Stress Scale (NSS)*: The NSS was developed by Ceylan and Bolışık (2017) to assess the level of stress in premature infants. Opinion about the use of the scale in term infants was obtained from one of the scale developers, Assoc. Prof. Dr. Sibel Serap Ceylan, via e-mail. In this three-point Likert-type scale, items are grouped into eight subgroups: facial expression, body color, respiration, activity level, comfort, muscle tone, extremities, and posture. In scoring, each subgroup is evaluated between 0 and 2 points. A minimum score of 0 and a maximum score of 16 points are obtained from the scale. A high score on the scale shows increased stress levels in the baby. Cronbach’s alpha coefficient was reported to be between 0.65 and 0.81 [24].

### Intervention

Applications made by the researcher during the data collection process; (1) swaddling of newborns, (2) assessing stress levels, (3) collecting saliva samples, (4) recording vital signs.

#### nCPAP Application Method

nCPAP application was performed using a Hamilton c1 neo device. Air leakage, circuit, and oxygen sensor control tests were performed before the application. Before the baby was connected to the ventilator, an interface (nasal tube 50, 70, 100 mm) and a binasal cannula or nasal mask (S, M, L) in sizes suitable for the baby’s weight were selected at the end of the ventilator set and connected to each other and worn on the newborn’s head. A sponge was placed in the area where the hat [appropriate size for the head circumference (17–22, 22–25, and 25–29 cm)] meets the nasal tube to reduce the risk of compression on the baby’s head, and the circuit connection was established. An orogastric catheter was inserted into the newborn to prevent gastric distension, and gastric decompression was provided by leaving the tip of the catheter open.

#### Swaddling Method

The newborn was placed in the supine position on a fabric blanket, naked except for a diaper; the upper edge of the fabric blanket was aligned with the newborn’s shoulder, the newborn’s arms were placed in the flexion-adduction position, and the horizontal ends of the fabric blanket were folded in the opposite direction to cover the upper trunk [25]. The baby’s head was allowed to move freely. The upper torso was completely wrapped with the fabric blanket. Then newborns were wrapped with the fabric blanket legs in flexion and abduction a suitable space was left at the bottom of the fabric blanket for the comfort of the newborn’s feet, and it was ensured that it was not too tight (17, 18). This whole swaddling process was completed in approximately one minute. A heat probe was placed in the axillary region before swaddling and for preventing hyperthermia the body temperature of the newborns was kept between 36.6 and 37.5 ºC [19, 26]. In the NICU, the incubator temperature is kept at 33.3 ± 1.0 °C in 1,500–2,500-gram babies and 32.8 ± 1.5 °C in 2,500-gram and above babies. Cotton, a soft textured fabric that does not irritate the skin of the newborn, was used for swaddling. The fabric blanket was square, 1800 cm^2^ (90 × 90 cm), and 75 g.

#### Collection of Saliva samples

In newborns, cortisol production fluctuates during sleep-wake cycles. In particular, cortisol levels decrease while the infant sleeps [27, 28]. Stressors, including painful stimuli, cause cortisol to rise. Cortisol levels peak approximately 20–30 min after an infant is exposed to an acute stressor. In preterm infants, the daily rhythm of cortisol production does not occur until at least one month of age [22, 29, 30]. Cortisol is secreted in a pulsatile way in a circadian rhythm in term infants from one month [22]. Therefore, saliva samples were collected in the study without regard to the morning-evening cycle.

It has been reported in the literature that saliva samples can be stored at -20 degrees for a maximum of 28 days [31] and at -80 degrees for one year [32]. Saliva samples collected in the study were stored at -20 degrees for approximately 14 days and at -80 degrees for approximately 4 months. Additionally, the study team included a researcher who had previously studied salivary cortisol levels.

Saliva samples of the newborns were collected using the SalivaBio Children’s Swab, which was specially prepared for this procedure. Saliva samples were collected at least 30 min after the completion of oral feeding of the infants in order to prevent the saliva sample from being mixed with milk [33]. In this process, after removing the cotton roll from the tube, it was held at one end and placed under the newborn’s tongue or in the corners of the mouth (2.5 cm) where saliva could accumulate. The cotton was allowed to become saturated with saliva for approximately 2 min. The cotton roll was held at the dry end and the wet part was placed back into the tube and the cap of the tube was closed. A barcode with the newborn’s name was affixed on the tube containing the saliva sample. The saliva sample was centrifuged at 1500 g for 15 min within 30 min after saliva collection [34]. The main purpose of centrifugation is to precipitate the saliva samples in the absorbent swab. All samples were stored at -80 °C until the number of samples (*n* = 40) was completed [35, 36].

### Pre-intervention

The newborn was placed in a supine position to better observe their chest movements and respiratory pattern. In order to ensure the patency of the airway of the newborn, who was connected to nCPAP, the newborn’s neck was supported with the help of rollers and placed in a slight extension position. At the 30th minute, when nCPAP, which is a stressful application, was started, the NSS was filled out by the researcher, and the first saliva sample was taken after vital signs were taken and recorded in the patient follow-up Tables [3, 21, 37].

### Post-intervention

Before the intervention, the newborn was monitored in the supine position connected to nCPAP and the slight extension position of the neck was maintained to ensure airway patency. After the first saliva sample was taken, the newborn was swaddled. In the swaddling procedure, the whole body of the newborn, who was laid on a fabric blanket with their legs in flexion and abduction position, was loosely swaddled. After 30 min of swaddling, the NSS was filled out by the researcher, vital signs were taken and the second saliva sample was taken from the newborn after being recorded in the patient follow-up Tables [3, 21, 34, 37].

#### ELISA Study

All samples tested in a blinded manner. Saliva-specific Enzyme-Linked ImmunoSorbent Assay (ELISA) was used for the study. The ELISA kit (DRG International, USA) was purchased commercially. User instructions were followed for kit contents. Standard and sample wells were measured in a spectrophotometer (ThermoScientific, USA) at 450 nm wavelength. Total concentration was calculated from the absorbance values for the standard and samples and cortisol levels were given as µg/dL.

### Data analysis

#### Sample size

In the study, the minimum sample size was calculated as 30 as a result of repeated measures analysis of variance with 80% power and alpha = 0.05 using the G Power 3.0.10 program in the known population (annual number of newborns in the said hospital) [38]. In the post hoc analysis based on pre-intervention and post-intervention NSS score averages, in the study was calculated as the effect size of 0.83 and 100% power (p < 0.001). In the post hoc analysis based on pre-intervention and post-intervention salivary cortisol levels average, in the study was calculated as the effect size 0.15 and 72% power (p = 0.013).

### Statistical analysis

IBM SPSS Statistics 25.0 (IBM SPSS Statistics for Windows, Version 21.0. Armonk, NY: IBM Corp.) package program was used for data analysis and numerical data were presented as mean, standard deviation, and minimum-maximum. In statistical analyses, the Shapiro-Wilk test was used to test whether the data fit the normal distribution. In the comparison of means in the dependent groups, a *t-*test was used because the data were normally distributed. The threshold level of statistical significance was *p* < 0.05 [39].

## Results

The mean gestational age of the newborns was 36.93 ± 1.50 (min. 35, max. 41) weeks and the mean maternal age was 29.07 ± 5.23 (min.17, max. 41) years. The mean age of the newborns was 2.10 ± 1.48 (min.1, max.5) hours, and the mean 1st- and 5th-minute APGAR scores were 8.30 ± 0.96 (min. 6, max. 9) 9.40 ± 0.90 (min.7, max. 10), respectively (Table 1).

**Table 1:** The Newborns’ Descriptive Characteristics

Variables	Mean ± *SD*	Min	Max
Week	36.90 ± 1.50	35	41
Mother’s age	29.07 ± 5.23	17	41
Age (hour)	2.10 ± 1.48	1	5
APGAR score (min 1)	8.30 ± 0.96	6	9
APGAR score (min 5)	9.40 ± 0.90	7	10
Birth weight (gr)	2,829.12 ± 522.88	2,000	4,410
Birth height (cm)	48.17 ± 2.09	43.00	53.00
Head circumference (cm)	33.25 ± 1.42	30.00	35.00
	***n***	**%**	
**Gender**			
Female	14	35	
Male	26	65	
**Delivery type**			
Vaginal	20	50	
Caesarean	20	50	
**nCPAP application method**			
Nasal cannula	13	32.5	
Nasal mask	27	67.5	

The mean birth weight of the newborns was 2,829.12 ± 522.88 (min. 2,000, max. 4,410) g, birth height was 48.17 ± 2.09 (min.43, max.53) cm, and birth head circumference was 33.25 ± 1.42 (min. 30, max. 35) cm. In the study, 65% of the newborns were male and 50% were born vaginally. Nasal cannula was used in 32.50% of the newborns and the nasal mask was used in 67.50%. (Table 1).

**Fig. 2:**
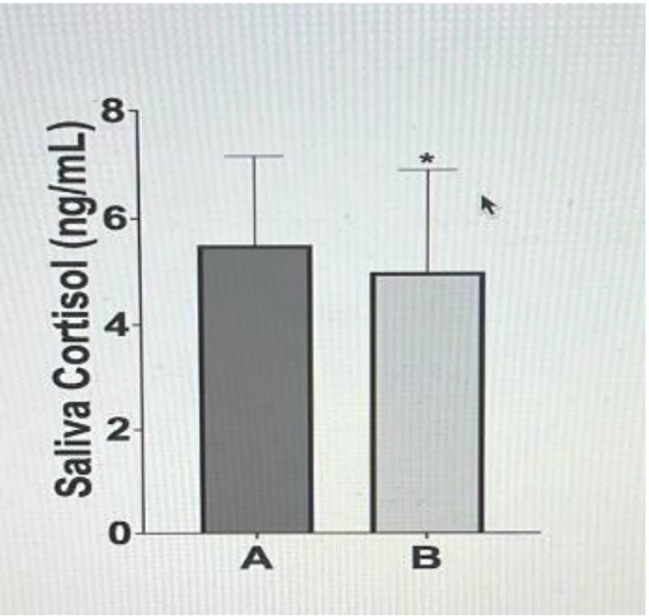
Pre and post-intervention saliva cortisol levels

A statistically significant difference was found between the mean NSS scores of the newborns before and after the intervention (*p* < 0.001). In addition, there was a statistically significant difference between the mean saliva cortisol levels of the newborns before and after the intervention (*p* < 0.05; Fig. 2; Table 2).

**Table 2:** Distribution of NSS Score and the Mean Saliva Cortisol Levels

Variables	Before intervention (nCPAP min 30)			After intervention (nCPAP min 60)			*t*-test/ *p-value*
	Mean ± *SD*	Min	Max	Mean ± *SD*	Min	Max	
NSS score	10.02 ± 2.05	6	14	3.52 ± 2.57	0	9	13.569/ ***p*** **< 0.001**
Saliva cortisol level (ng/Ml)	5.51 ± 1.65	1.22	7.41	4.99 ± 1.89	0.74	7.45	2.614/ **0.013**

A statistically significant difference was found between the mean heart rate of the newborns before and after the intervention (*p* < 0.05). The difference between the mean oxygen saturation of the newborns before and after the intervention was not statistically significant (*p* = 0.292). Additionally, there was a statistically significant difference between the mean respiratory rate of the newborns before and after the intervention (*p* < 0.001; Table 3).

**Table 3:** Distribution of Vital Signs of Newborns

Variables	Before intervention (nCPAP min 30)			After intervention (nCPAP min 60)			*t*-test/ *p-value*
	Mean ± *SD*	Min	Max	Mean ± *SD*	Min	Max	
Heart Rate (min)	140.82 ± 18.11	96	175	135.50 ± 14.15	100	173	2.38/ **0.022**
Oxygen Saturation	98.05 ± 3.35	85	100	98.62 ± 1.82	92	100	-1.06/ 0.292
Respiratory rate (min)	72.95 ± 9.06	56	92	68.07 ± 10.16	54	95	4.74/ ***p*** **< 0.001**

## Discussion

In this part of the study examining the effect of swaddling method on stress levels in newborns receiving nCPAP, NSS scores, salivary cortisol levels and vital signs before and after the intervention were discussed.

The study found that the mean NSS score of the newborns at the 30th minute after the swaddling method was applied was lower than the 30th minute before the intervention (*p* < 0.05). Apaydin Cirik and Efe (2020) reported that the combined method of swaddling + breast milk significantly reduced the premature infant pain profile (PIPP) score of preterm newborns during orogastric catheter insertion compared to other groups (whom routine care, swaddling, facilitated tucking, expressed breast milk, facilitated tucking + expressed breast milk were applied), and the pain score of newborns whom swaddling method was applied was lower than the routine care group (*p* < 0.001) [11]. A previous study found that newborns who underwent the swaddling method during heel prick blood collection had a lower mean NIPS score during the procedure than newborns who were placed supine on the procedure able [16]. Another study reported that three different methods (swaddling, swaddling + holding, and swaddling + holding + breastfeeding) played a role in reducing the pain felt during the heel prick procedure and that the mean NIPS score was lower in newborns who underwent the swaddling method compared to the control group (supine position) [17]. Another study found that two different methods (swaddling and holding) were effective in reducing the pain felt during the heel prick procedure in newborns, and the mean NIPS score was lower in newborns who underwent the swaddling method compared to the control group (supine position) (*p* < 0.05) [18]. In the study conducted by Ho et al. (2016), the mean PIPP score of preterm infants who underwent the swaddling method during the heel prick procedure was lower than the control group who were given the supine position [15]. According to the results of this study, the swaddling method applied during invasive interventions (Orogastric (OG) insertion, heel prick, etc.) applied to newborns plays a role in reducing the pain level of newborns and also the stress level they experience. To date, no investigation has examined the effect of the swaddling method on the stress level of newborns undergoing nCPAP. It is thought that the swaddling method is instrumental in reducing the stress experienced by newborns undergoing nCPAP due to respiratory distress.

In the present study, the saliva cortisol levels in the 30th minute of the swaddling method during nCPAP applied to newborns were lower than that in the first 30 min during which no intervention was applied (*p* < 0.05). No study examining the effect of the swaddling method on saliva cortisol levels in newborns receiving nCPAP was found in the literature; it is thought that the swaddling method also plays a role in reducing the stress experienced by newborns diagnosed with TTN and receiving nCPAP due to respiratory distress.

This study found that the mean scores of heart rate and respiratory rates in the 30th minute of the swaddling method during nCPAP applied to newborns were lower than those in the 30th minute before the intervention without any intervention (*p* < 0.05). Ho et al. (2016) reported that the heart rate of newborns to whom the swaddling method was applied during the heel prick procedure was higher than that of the control group, and it was lower than the control group immediately after the heel prick procedure [15]. It was reported in the study of Apaydin Cirik and Efe (2020) that the mean heart rate during the OG insertion procedure was lower in the swaddling group compared to the routine care group and other groups (swaddling + expressed breast milk, facilitated tucking, expressed breast milk, and facilitated tucking + expressed breast milk) [11]. In the study of Huang et al. (2004), there was no difference between the heart rates of premature newborns who were swaddled during the heel prick procedure and those who were given the fetal position; however, the premature babies who were swaddled returned to the initial heart rate in a shorter time [14]. The results of the studies which reported that the swaddling method was effective in decreasing respiratory and heart rate, an indicator of the stress experienced by newborns due to pain [11, 14, 15], are consistent with the results of this present study. In this study, it is thought that swaddling played a role in decreasing the respiratory rate and heart rate of newborns during nCPAP, which were high in the 30th minute before the intervention.

### Limitations of the study

The limitations of the study were that it was not a randomized-controlled study, newborns under 35 weeks of age were not included, and only infants with TTN were included in the study.

The time of hospitalization of the newborns included in the sample group to the clinic was variable; therefore, the study data were collected at different times of the day, which was the difficulty experienced in the study. Accordingly, the samples were collected by the researcher to ensure that the data were collected according to the same standards. Since the samples were collected at different times of the day, the data were also collected outside of working hours. In addition, these samples were temporarily stored at -20 ^o^C in the Blood Bank laboratory of the city hospital, where the research was conducted to prevent the deterioration of the samples. Every two weeks, the samples were transferred to Kütahya Health Science University, Central research laboratory, where the analyses would be performed, for storage at -80 ^o^C in accordance with the cold chain conditions.

Since the research was a master’s thesis, there was a time constraint. Therefore, gestational age could not be held constant. Since the baby was receiving treatment in the neonatal intensive care unit, the mother’s stress level could not be measured because it was not possible for the mother and baby to be together. Since the days and hours worked by the nurses in the neonatal intensive care unit, where the research was conducted, were not fixed, a second observer could not be included in the study.

## Conclusions

Based on the results of this study, the swaddling method plays a role in the reduction of the NSS score and saliva cortisol levels. In this context, H1 and H2 hypotheses were accepted. It was concluded that the swaddling method applied to newborns decreased heart rate and respiratory rate, but it had no effect on oxygen saturation. In conclusion, randomized controlled trials, including long-term follow-ups of the effect of swaddling on relieving stress and pain in newborns on nCPAP in NICUs, should be conducted. The swaddling method should be used to reduce the stress level of newborns during nCPAP, and this method should be included in the care protocols to be established for nCPAP application.

## Data Availability

The datasets used and analyzed during the current study are available from the corresponding author on reasonable request.

## Abbreviations

nCPAP: Nasal Continuous Positive Airway Pressure
TTN: Transient Tachypnea of newborn
BPD: Bronchopulmonary Dysplasia
RDS: Respiratory Distress Syndrome
MAS: Meconium Aspiration Syndrome
NICU: Neonatal Intensive Care Units
NSS: Newborn Stress Scale
PIPP: Premature Infant Pain Profile
OG: Orogastric
